# Ginseng Polysaccharide Enhances the Humoral and Cellular Immune Responses to SARS-CoV-2 RBD Protein Subunit Vaccines

**DOI:** 10.3390/vaccines11121833

**Published:** 2023-12-08

**Authors:** Jing Zhang, Jing Feng, Yang Huang, Boyan Zhou, Bing Li, Rongxin Zhang

**Affiliations:** Laboratory of Immunology and Inflammation, School of Life Sciences and Biopharmaceutics, Guangdong Pharmaceutical University, Guangzhou 510006, China; 1112342005@gdpu.edu.cn (J.Z.); 2112242008@gdpu.edu.cn (J.F.); 1700903104@gdpu.edu.cn (Y.H.); 2112342045@gdpu.edu.cn (B.Z.)

**Keywords:** ginseng polysaccharide, SARS-CoV-2, RBD protein, vaccine, adjuvant, humoral immunity, cellular immunity

## Abstract

The COVID-19 pandemic remarkably accelerated vaccine research progress. The role of adjuvants in enhancing vaccine immune intensity and influencing immune types has been considered. Ginseng polysaccharide (GPS) has been demonstrated to have strong immunoregulatory properties. It is important to explore the feasibility of adding GPS to vaccine adjuvant components to improve the immune response effect of RBD vaccines. Here, we prepared a SARS-CoV-2 RBD antigen using the *Escherichia coli* expression system and determined that subcutaneous administration of GPS at a dose of 40 mg/kg could effectively activate dendritic cells (DCs) and macrophages (MΦ) in mice. Compared with the RBD group, the RBD+GPS triggered stronger and persistent antibody responses. It is also notable that higher levels of RBD-specific IgG and IgA were distributed in the lungs of RBD+GPS-immunized BALB/c mice. In addition, the RBD+GPS also resulted in lower percentages of IFN-γ^+^ CD4^+^ T cells and higher percentages of IFN-γ^+^ CD8^+^ T cells and CD8^+^ Tcm cells. These results suggest that GPS could be a promising vaccine immuno-enhancer for SARS-CoV-2 RBD subunit vaccines to establish stronger systemic and pulmonary mucosal protective immunity.

## 1. Introduction

COVID-19 is a highly infectious respiratory disease that caused a global pandemic and severely damaged both human health and economic development [1]. The most valid method to prevent the development of SARS-CoV-2 is vaccination, and various types of COVID-19 vaccines, including inactivated vaccines, viral vector vaccines, mRNA vaccines, and subunit vaccines have emerged as the circumstances required [2]. Among them, subunit vaccines have higher safety and wider applicability. As the essential component in subunit vaccines, an adjuvant is an immunostimulant that not only enhances the strength and longevity of immunity but also influences the type of response. Aluminum salts, AS03, MF59, matrix-M adjuvant, and CpG have been applied to COVID-19 subunit vaccines to enhance the immune response [3,4,5,6]. However, these adjuvants could improve humoral responses and immunologic memory but failed to induce the cytotoxic T cell response and mucosal immune system [7]. Therefore, it is necessary to explore and research new bioactive substances with immune-enhancing effects to enhance the immune response to COVID-19 vaccines.

Traditional Chinese medicine (TCM) has a long and profound history. The commonly used TCM can be divided into 18 categories and 364 types, and the prescriptions obtained through different herbal combinations are countless. With the development of extracting technology and analyzing methods, the effective ingredients and safety of TCM have been researched deeply. Previous studies showed that TCM’s effective ingredients (including polysaccharides, saponins, flavonoids, terpenoids, and so on) exert antiviral functions by directly inhibiting the virus invasion of cells, virus replication, assembly, maturation, and release [8,9,10,11]. More than that, TCM’s effective ingredients have a significant immunomodulatory function, gaining more and more attention as vaccine adjuvants, especially polysaccharide components, such as *Astragalus* polysaccharides, *Ganoderma lucidum* polysaccharides, *Angelica sinensis* polysaccharides, *Codonopsis pilosula* polysaccharides, and so on, which have been reported to enhance the immune effect of hepatitis B virus vaccine, influenza vaccine, porcine circovirus vaccine, foot-and-mouth disease vaccine, and porcine reproductive and respiratory syndrome virus vaccine [12].

As a famous TCM, ginseng plays an important role in the treatment of various diseases. Ginseng contains multiple active ingredients, such as ginsenosides, ginseng polysaccharides (GPS), amino acids, vitamins, volatile oils, and more than 17 trace elements [13]. GPS and ginsenosides have always been considered the main bioactive components, which can exert anti-tumor, immune regulation, antiviral, antioxidant, and anti-inflammatory effects [14,15]. Results reported by Shin showed that GPS can exert antimetastatic activity by promoting macrophages(MΦ) and NK cell activation [16]. Ginseng crude extract or its ingredients also exert an immunoadjuvant function, which could enhance Th1- or Th2-type immune responses against protein or bacteria antigens [17,18]. Adding GPS to inactivated porcine parvovirus and using aluminum salt as an adjuvant to immunize pigs could induce higher levels of serum antibody titers [19]. However, the specific function of GPS as an immune adjuvant has not been thoroughly studied. Here, we found GPS can effectively activate dendritic cells (DCs) and MΦ to enhance the ability of RBD antigens to initiate immune responses. We further demonstrated that GPS-assisted SARS-CoV-2 RBD subunit vaccines elicited strong and long-lasting humoral immunity but also triggered CD8^+^ T cell and Th1-biased CD4^+^ T cell responses. The present study revealed the potential of GPS as an immune adjuvant to improve the immune efficiency of SARS-CoV-2 RBD subunit vaccines or other infectious disease vaccines.

## 2. Materials and Methods

### 2.1. Animals

Female BALB/c mice, 6 weeks old, were provided by the Medical Experimental Animal Center of Guangdong Province, and all the procedures used during the research were approved by the Local Ethics Committee for Animal Testing at the Guangdong Pharmaceutical University, China (approval number gdpulac2021069). The GPS+RBD group of mice (*n* = 20, the following per group is consistent) were subcutaneously immunized with 10 μg doses of RBD recombinant protein and 40 mg/kg doses of GPS (HPLC ≥ 90%, Vickqi Biological Co., Ltd., Chengdu, China) formulated with Sigma Adjuvant System (SAS) adjuvant. The SARS-CoV-2 RBD subunit vaccine group of mice was immunized with equal moles of RBD recombinant protein. Meanwhile, the control group of mice was immunized with equal volumes of the PBS and adjuvant. All the mice were initially immunized at week 0 and booster immunized at weeks 2 and 4. The eyeball blood of mice was collected at weeks 4, 6, 10, 14, and 18.

### 2.2. Protein Expression and Purification

The RBD DNA sequences with 6 × His-tags were cloned into a pET-28a vector. The recombinant expression vector was converted to *Escherichia coli* (*E.coli*) BL21 (DE3) (Sangon Biotech, Shanghai, China). After induction for 5 h under 0.8 M IPTG at 37 °C while shaking, the cultures were collected and ultrasonically crushed. The RBD recombinant protein expressed in precipitates was dissolved in 8 M urea and purified with Ni-TED Sefinose Resin 6FF (Sangon Biotech, Shanghai, China). The purified protein was concentrated through ultrafiltration tubes and renatured by using PBS buffer. The results of the protein expression and purification were analyzed by Coomassie blue staining.

### 2.3. BAL Collection

After the mice were euthanized and exsanguinated (*n* = 3 per group at each time point), the lungs were slowly washed by using 1 mL of mucosal preparation solutions (0.01 M pH 7.4 PBS, 0.01% protease inhibitor, 0.05% sodium azide, and 0.05% Tween-20) with a 20 G blunt-ended needle. The bronchoalveolar lavage fluid (BAL) was collected and centrifuged at 350× *g* for 8 min. The supernatants were stored at −80 °C and used for the anti-RBD IgG and IgA ELISA detection. The titers of BAL antibodies against IgG and IgA were analyzed using GraphPad Prism 8.4.2 software.

### 2.4. Enzyme-Linked Immunosorbent Assay

The high-binding 96-well plates were coated with 4 μg/mL of recombinant RBD protein and then incubated overnight at 4 °C. The liquid in the 96-well plates was discarded and, using 5% milk in PBST, the plates were blocked at 37 °C for 90 min. After serial dilutions of immunized mouse serum samples and BAL samples, triplicates of each were added to 96-well plates and then incubated for 1 h at 37 °C. After washing the well plates three times with PBST, the primary antibody was added to each well, and the incubation was continued for 1 h at 37 °C. The antibodies used were HRP-conjugated anti-mouse IgG (BBI, Shanghai, China) and HRP-conjugated anti-mouse IgA (Proteintech, Rosemont, IL, USA). After effective integration, the color reaction was carried out with TMB solution and aborted with termination solution. Finally, the absorbance was read at 450 nm using a microplate reader (Bio-Te, Irving, TX, USA). All the ELISA data were analyzed using GraphPad Prism 8.4.2 software.

### 2.5. Lymphocyte Preparation

The inguinal lymph nodes and spleens were harvested after the BALB/c were euthanized (*n* = 3 per group at each time point), mechanically ground, and filtered with a 70 μm cell filter (BD, New York, NY, USA) to prepare single-cell suspensions. After centrifugation, the spleen cells were fragmented with red blood cell lysis buffer (Solarbio, Beijing, China) for 15 min. Then, using 10 mL of PBS-terminated red blood cell lysis, the cells were resuspended once in 5 mL of PBS prior to being used for the flow cytometric and ELISpot analyses.

### 2.6. IFN-γ Enzyme Linked Immunospot Assay

Enzyme-linked immunospot (ELISpot) assays were performed using the mouse IFN-γ precoated ELISpot kit (Dakewe Biotech Co., Ltd., Shenzhen, China). The IFN-γ precoated ELISpot plate was activated by serum-free medium (Dakewe Biotech Co., Ltd., Shenzhen, China). Then, different groups of mice spleen cells were inoculated into each well and stimulated with the SARS-CoV-2 RBD Peptide 1: VGYQPYRVVVLSFEL or Peptide 2: VRQIAPGQTGKIAD (synthesized by GenScript) at concentrations of 0.1 mg/well for 18–22 h. The liquid in the 96-well plates was discarded, and ice-cold aqua pura lysed the residual cells. The 96-well plate was incubated with antibodies and colored according to the manufacturer’s instructions. The spots were scanned and quantified by an ImmunoSpot Mabteh IRIS reader. The number of spots was plotted as the mean ± SEM (Standard Error of the Mean) using GraphPad Prism 8.4.2 software.

### 2.7. Flow Cytometry

At different time points, the inguinal lymph nodes and spleens were mechanically ground to prepare lymphocytes from the RBD- or GPS+RBD-immunized mice. To avoid non-specific binding, the collected cells were blocked with anti-CD32 /CD16 (BioLegend) after a single wash. The lymphocytes were stained using the following antibodies: FITC-anti CD4 (1:100), APC-anti IgD (1:100), APC-anti CD8a (1:100), APC-anti CXCR5 (1:100), BV421-anti CD19 (1:100), PE-Cy7-anti CD62L (1:100), PE-Cy5.5-anti CD4 (1:100), PE-anti CD44 (1:100), APC-Cy7-anti CD45R (1:100), BB515-anti CD3e (1:00), PE-anti CD95 (1:100), PE-anti PD-1 (1:100), PE-anti GL7 (1:100), and FITC-anti CD38 (1:100). To evaluate the effects after vaccination, the samples were measured by the flow cytometer, Celesta (flow cytometric Canto II, BD Biosciences).

### 2.8. Intracellular Cytokine Staining

In order to evaluate the antigen-specific T cells to the RBD protein, a single cell suspension was prepared from the spleen of the mice and stimulated in a 96-well plate, using eBioscience Cell Stimulation Cocktail (Invitrogen, Waltham, MA, USA) at 37 °C with 5% CO_2_ for 8–12 h. The cells were processed by a fixation/permeabilization kit (BD Biosciences, Franklin Lakes, NJ, USA), followed by staining with IL-4 (BD), IFN-γ, and TNF-α (BioLegend, San Diego, CA, USA). The following cytokine antibodies were used: PE-Cy7-anti IL-4 (BD), PE-anti IFN-γ (BioLegend), and APC-anti TNF-α (BioLegend). The proportion of IFN-γ^+^ and TNF-α^+^ CD8^+^ T cells and memory T cells were analyzed by flow cytometry (BD Biosciences, Franklin Lakes, NJ, USA) and FlowJo software (version 10.6.2).

### 2.9. Statistical Analysis

All the values are expressed as the mean ± SEM (Standard Error of the Mean). Antibodies titers, ELISpot data, and cytokine levels were evaluated using one-way ANOVA or *t*-tests. All the statistical analyses were conducted utilizing Graphpad Prism 8.4.2. The data were considered significant if the *p*-value < 0.05.

## 3. Results

### 3.1. Ginseng Polysaccharide-Assisted RBD Protein More Effectively Initiated Immune Response

**Figure 1:**
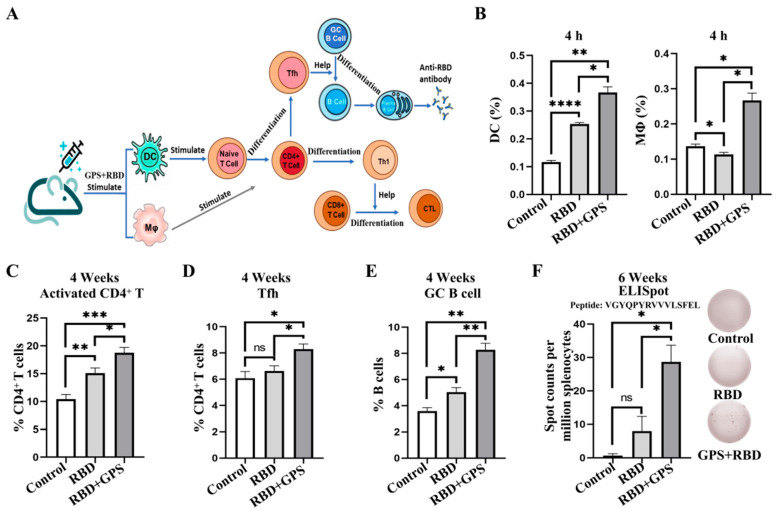
Ginseng polysaccharides-assisted RBD protein more effectively initiated immune response. (**A**) The GPS and RBD proteins act as antigens for immunization, then activate DCs and MΦ. DCs promote naïve T cell differentiation. (**B**) The BALB/c mice were immunized subcutaneously with the RBD proteins and the GPS+RBD, which were adjuvanted with SAS adjuvant, respectively. At 4 h postinjection, inguinal lymph nodes were obtained for the flow cytometric analysis to detect the levels of DCs and MΦ (*n* = 3). (**C**–**E**) 4 weeks post-immunization, the mice immunized with different antigens were euthanized. The percentages of activated CD4^+^ T cells, Tfh cells, and GC B cells within the spleens were determined by using flow cytometric analysis (*n* = 3). (**F**) 6 weeks post-immunization, IFN-γ^+^ T cells were determined by using ELISpot. The experiments were conducted independently in triplicate. The data are expressed as the means ± SEMs (Standard Error of the Mean). ns—not significant, * *p* < 0.05, ** *p* < 0.01, *** *p* < 0.001, and **** *p* < 0.0001.

As the most potent antigen-presenting cells (APCs), DCs activate naïve T cells to transform into effector T cells for adaptive immune responses (Figure 1A). Meanwhile, MΦ are important APCs that present antigens to corresponding Th cells and play a crucial role in initiating immune responses. It has been shown that GPS is responsible for immunomodulatory functions, such as activating MΦ and T cells [20]. The RBD recombinant protein was expressed in *E.coli*, purified by using Ni-TED 6FF, and verified through Coomassie blue staining analysis (Supplementary Figure S1A,B). The concentration and mode of administration of GPS were determined in immune activation by flow cytometry (Supplementary Figure S1C–E). The result showed that subcutaneous delivery of GPS at a dose of 40 mg/kg could effectively activate DCs and MΦ in mice. The flow cytometric assay revealed a significant increase in the percentages of DCs and MΦ in the GPS+RBD immunization group compared to the RBD protein immunization group (Figure 1B). Furthermore, the percentages of follicular helper T (Tfh) cells and germinal center B (GC B) cells, which are important in the establishment of a humoral immune response [21], were significantly higher in the GPS+RBD group of immunized mice than in the RBD protein-vaccinated mice (Figure 1C–E). Meanwhile, the ELISpot results also indicated that the GPS+RBD induced stronger IFN-γ^+^ CD8^+^ T cell responses (Figure 1F). These results indicated that GPS could improve the recognition efficiency of DCs and MΦ to RBD proteins, thereby effectively initiating adaptive immune responses.

### 3.2. Ginseng Polysaccharides Enhanced Humoral Immune Responses in RBD Protein

**Figure 2:**
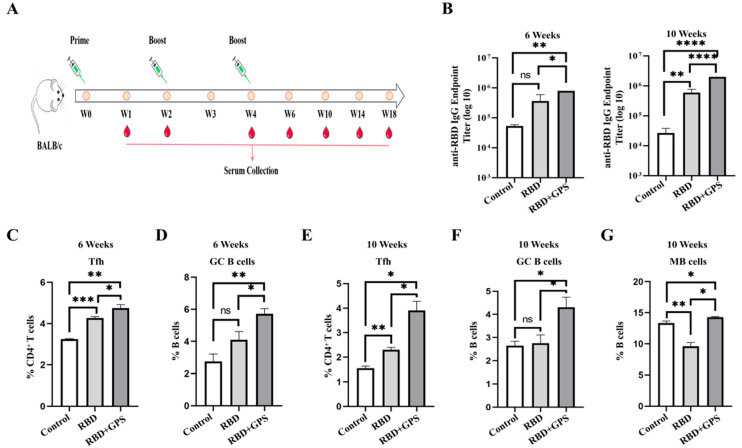
Ginseng polysaccharides enhanced humoral immune responses to RBD protein. The female BALB/c mice were injected with 10 μg of RBD protein and 40 mg/kg of GPS by hypodermic injection. (**A**) The BALB/c mice were immunized at week 0, week 2, and week 4 with the RBD protein and the GPS+RBD, and the serum samples were collected at week 1, week 2, week 4, week 6, week 10, week 14, and week 18. (**B**) The endpoint titers of the SARS-CoV-2 RBD-specific IgG antibodies in the serum were detected by ELISA in week 6 and week 10. (**C**–**G**) The percentages of Tfh cells, GC B cells, and memory B cells (MB) cells within the spleens were determined by using flow cytometric analysis (*n* = 3). The experiments were conducted independently in triplicate. The data are expressed as the means ± SEMs (Standard Error of the Mean). ns—not significant, * *p* < 0.05, ** *p* < 0.01, *** *p* < 0.001, and **** *p* < 0.0001.

The humoral immune response of the GPS+RBD group was estimated in the BALB/c mice, and equal moles of RBD protein and GPS were subcutaneously injected three times in a prime-boost manner (Figure 2A). The RBD-specific IgG titers in the GPS+RBD group rapidly rose to 10^5^ in week 6, and to 10^6^ in week 10 post-prime immunization, but the titers of the RBD group were induced to below 10^6^ in week 6 and week 10 (Figure 2B). Compared with the RBD group, higher percentages of Tfh, GC B, and MB cells were also detected in the GPS+RBD group 10 weeks after the initial immunization (Figure 2C–G and Supplementary Figure S2C). These results indicated that GPS could enhance the levels of RBD-specific IgG and more Tfh, GC B, and MB cells, which could provide persistent humoral responses.

### 3.3. Pulmonary Biodistribution of SARS-CoV-2 RBD Reactive IgG and IgA

**Figure 3:**
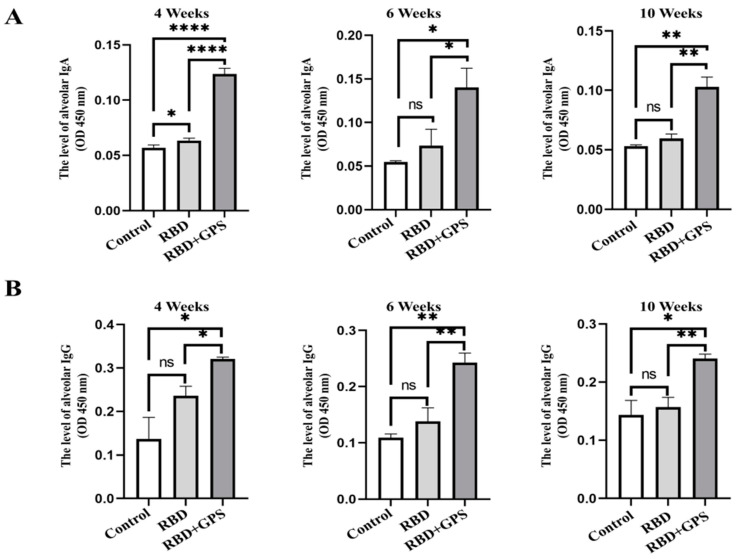
Biodistribution of SARS-CoV-2 RBD-reactive IgG and IgA in the lungs. Detection of the severe acute respiratory syndrome coronavirus 2 (SARS-CoV-2) RBD protein-reactive antibodies in BAL samples (1:50 dilution). (**A**) The level of the SARS-CoV-2 RBD-specific IgA antibodies in the BAL were detected by ELISA in week 4, week 6, and week 10. (**B**) The level of the SARS-CoV-2 RBD-specific IgG antibodies in the BAL were detected by ELISA in week 4, week 6, and week 10. The RBD-specific IgA and IgG levels were detected at OD 450 nm. The experiments were conducted independently in triplicate. The data are expressed as the means ± SEMs (Standard Error of the Mean). ns—not significant, * *p* < 0.05, ** *p* < 0.01, and **** *p* < 0.0001.

The mucosal immune system is the primary defensive line in the human body against respiratory infectious diseases, and the IgG and IgA antibodies on the pulmonary mucosa can neutralize viral infection activity. Hence, SARS-CoV-2 RBD-specific IgG and IgA in the bronchoalveolar lavage (BAL) fluid from the immunized mice were evaluated at week 4, week 6, and week 10 after the initial immunization. Compared with the RBD protein group, the GPS+RBD group had a statistically significant increase in SARS-CoV-2 RBD protein-binding IgA and IgG (Figure 3A,B). These data demonstrate that GPS+RBD can induce more anti-SARS-CoV-2 RBD-specific IgG and IgA antibodies to reduce the possibility of virus infection.

### 3.4. GPS Improved RBD Protein Cellular Immune Responses in Mice

**Figure 4:**
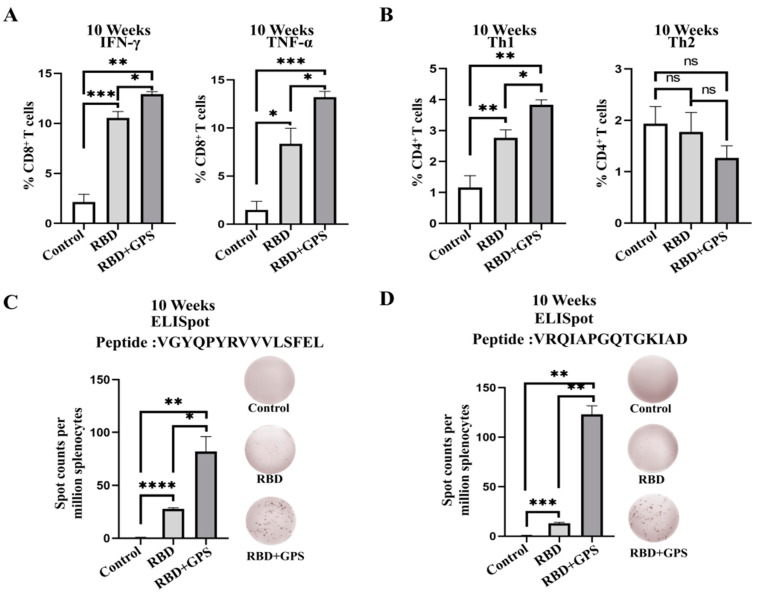
T cell immune responses in GPS+RBD-vaccinated BALB/c mice. The GPS initiated higher levels of T cell immune responses to the SARS-CoV-2 RBD protein in BALB/c mice. The BALB/c mice were euthanized to harvest their splenocytes for ICCS and ELISpot assays. (**A**) In week 10, the percentages of IFN-γ^+^ and TNF-α^+^ CD8^+^ T cells were determined by ICCS (*n* = 3). (**B**) The percentages of IFN-γ^+^ and IL-4^+^ CD4^+^ T cells were determined by ICCS (*n* = 3). (**C**,**D**) The splenocytes were stimulated with different S peptide pools. ELISpot assays were conducted for IFN-γ secretion in the splenocytes. The experiments were conducted independently in triplicate. The data are expressed as the means ± SEMs (Standard Error of the Mean). ns—not significant, * *p* < 0.05, ** *p* < 0.01, *** *p* < 0.001, and **** *p* < 0.0001.

Cellular immunity is the key to regulating disease severity and eliminating infection [22]. To determine whether the GPS+RBD could induce strong T cell responses, groups of BALB/c mice were executed at week 10 post-prime immunization, and their splenocytes were prepared for ICCS and T cell IFN-γ ELISpot analysis. The analysis results of the ICCS showed that the proportion of IFN-γ^+^ CD8^+^ T cells and TNF-α^+^ CD8^+^ T cells in the GPS+RBD group was higher than that in the RBD protein group (Figure 4A and Supplementary Figure S2C). A previous study on SARS-CoV revealed that Type 1 T helper cell (Th1)-biased immune responses enhanced protection against virus infection, but Type 2 T helper cell (Th2)-biased immune responses may lead to the occurrence of Th2-type immunopathology [23]. Therefore, the proportion of IFN-γ^+^ CD4^+^ T cells (Th1-biased cells) and IL-4^+^ CD4^+^ T cells (Th2-biased cells) were counted by ICCS. The higher level of Th1-biased IFN-γ^+^ CD4^+^ T cells were observed in the GPS+RBD group. No difference was found in the percentages of Th2-biased IL-4^+^ CD4^+^ T cells in all the groups (Figure 4B). The conserved peptides VGYQPYRVVVLSFEL [24] and VRQIAPGQTGKIAD [25] were used to perform the ELISpot assay, and higher percentages of IFN-γ^+^ CD8^+^ T cells were observed in the GPS+RBD group (Figure 4C,D). These results indicate that GPS+RBD can stimulate stronger and safer T cell immune responses.

### 3.5. Ginseng Polysaccharides Enhanced Long-Term Protective Memory in RBD Protein

**Figure 5:**
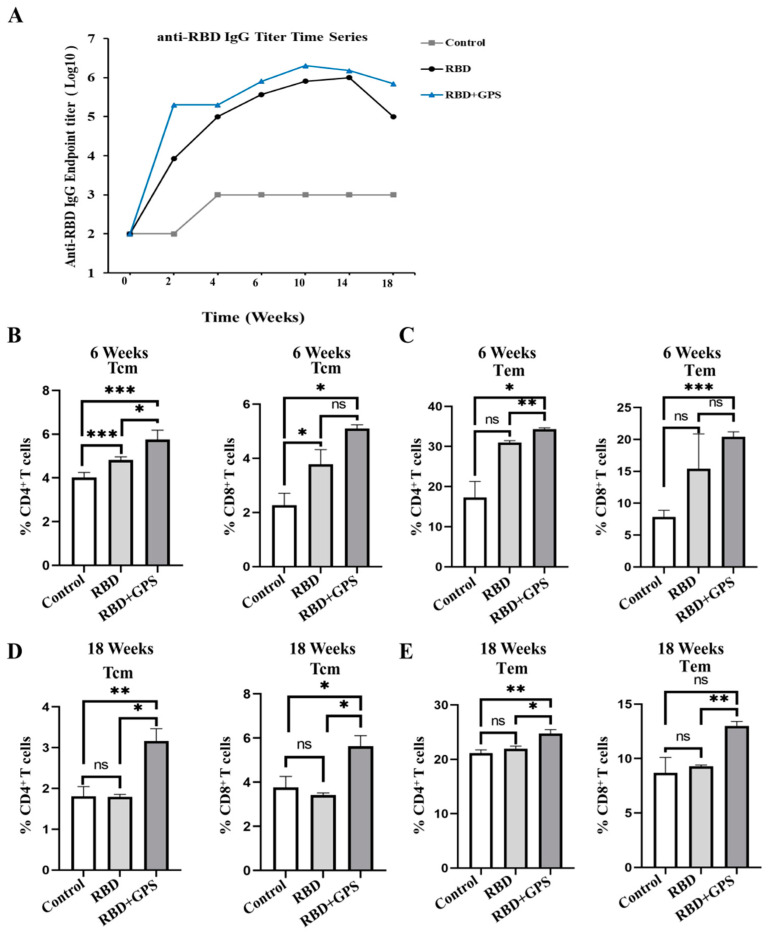
Ginseng polysaccharides enhanced long-term protective memory in RBD protein. (**A**) The SARS-CoV-2 RBD-specific IgG titers of the immunized BALB/c mice at each time point were detected by using ELISA. (**B**,**D**) The immunized mice were euthanized at 6 and 18 weeks post-prime vaccination to harvest their splenocytes, and the CD4^+^ Tcms and CD8^+^ Tcms were determined by using flow cytometric analysis (*n* = 3). (**C**,**E**) The immunized mice were euthanized at 6 and 18 weeks post-prime vaccination to harvest their splenocytes, and the CD4^+^ Tems and CD8^+^ Tems were determined by using flow cytometric analysis (*n* = 3). The experiments were conducted independently in triplicate. The data are expressed as the means ± SEMs (Standard Error of the Mean). ns—not significant, * *p* < 0.05, ** *p* < 0.01 and *** *p* < 0.001.

The maintenance of immune memory is a measure for evaluating vaccines’ efficacy. The antibody detection results showed that the RBD-specific IgG titer induced by GPS+RBD reached its peak at week 10, faster than the titer of the RBD protein group, which reached its peak at week 14. The titers of the RBD-specific IgG in the GPS+RBD group remained at 10^6^ at week 18 after initial vaccination and were maintained for a long time, but the titers in the RBD protein group decreased to 10^4^–10^5^ (Figure 5A). Furthermore, compared with the RBD group, the GPS+RBD induced higher levels of CD4^+^ and CD8^+^ central memory T (Tcm) and effector memory T (Tem) cells (Figure 5B–E), which contributed to modulating the disease severity [26]. Based on the above, GPS+RBD can induce higher and longer-lasting protective T cell immune responses.

## 4. Discussion

The public health event caused by the SARS-CoV-2 infection facilitated scientific research on vaccines. Over 90% of vaccines contain adjuvants, which play important roles in maintaining vaccines’ persistence and effectiveness and reducing adverse events to ensure safety [27]. Due to the absence of the entire pathogen or nucleic acid components, the safety of subunit vaccines is good, but with low immunogenicity so therefore need to be used in conjunction with adjuvants. Currently commonly used adjuvants, especially aluminum adjuvants, can effectively improve the level of humoral immune response but with limited activating ability on cellular immune responses [28]. With the development of TCM technology, the application of TCM extracts in immunotherapy is becoming increasingly widespread. Ginseng ingredients have anti-tumor, immune regulation, and antiviral effects [8,9,10]. Our research found the feasibility of GPS as an immuno-enhancer for SARS-CoV-2 subunit vaccines. The results indicated that GPS can activate DCs and MΦ to recognize RBD protein, initiating immune responses effectively, and further inducing high-level humoral and cellular immune responses in BALB/c mice. The humoral immune effector product antibodies (IgA and IgG) can directly neutralize the virus, and the GPS+RBD group induced higher levels of IgA and IgG in our research. The cellular immune effector product, IFN-γ^+^ CD8^+^ T cells, can clear the virus infecting somatic cells to promote body recovery, and the GPS+RBD group induced higher levels of IFN-γ^+^ CD8^+^ T cells. Meanwhile, GPS also improves RBD subunit protein safety and prolongs immune memory.

DC cells are the core part of the process of immune initiation, immune regulation, and maintenance of immune response [28]. GPS stimulates DCs to promote CD4^+^ T lymphocyte proliferation by secreting IFN-γ to activate MΦ, thus enhancing immunity [29]. Thus, this study found that the GPS concentration of activated DCs and MΦ in the subcutaneously immunized mice with RBD protein served as an adjuvant enhancer. The results confirmed that GPS can assist RBD protein in inducing DCs and MΦ production in subcutaneously immunized mice. An existing clinical study has shown that after 6 weeks of BNT162b2 or ChAdOx1 complete vaccination, the total antibody level in the body begins to decrease to more than 50% within 10 weeks [30]. The results of this study showed that the GPS+RBD group remained at 10^6^ at week 18 after initial vaccination and remained at a high titer level of RBD-specific IgG, which indicated that GPS has the potential to solve the issue of short maintenance time of antibody levels. Tfh cells are involved in the differentiation of GC B cells into MB cells and plasma cells. At the same time, these cells are closely related to the strength and duration of humoral immune response after antigen immunization [21]. Lower levels and short maintenance of memory B cells and serum IgA and IgG will lead to more serious illness and even death from COVID-19 [31]. The application of adjuvants may solve the problem of sustained antibody levels. According to the continuous monitoring of Tfh cells, GC B cells, MB cells, and IgG, the results were consistent with this supposition. The lung is the target organ of a SARS-CoV-2 attack, and the antibodies distributed in the lung can inhibit virus infection, especially IgA. A recent study showed that the lack of IgA defense would increase the risk of COVID-19 patients suffering from serious COVID-19 by 7.7-fold [32]. In this study, we observed that GPS could be used as an immuno-enhancer to significantly improve the levels of anti-RBD-specific IgA and IgG, not only in the serums but also in the lungs, which could contribute to reducing the probability of severe respiratory disease.

Cellular immune responses can potentially reduce the virus load and spread while facilitating faster recovery in SARS-CoV-2 [33,34]. Here, we found that GPS improved the capacity of RBD protein to induce much higher IFN-γ^+^ CD8^+^ and TNF-α^+^ CD8^+^ T cell responses. Although the S protein or RBD region is prone to mutation, causing the antibody to lose neutralizing activity, it still contains multiple conserved T cell epitopes in SARS-CoV variant coronaviruses, which could provide a cross-protection effect. The reactive CD8^+^ T cell epitopes, including VGYQPYRVVVLSFEL and VRQIAPGQTGKIAD, were used in the ELISpot assay. The results indicated that GPS could assist RBD protein in inducing higher IFN-γ^+^ CD8^+^ T cell responses, which targeted the above epitopes to remove the cells influenced with SARS-CoV-2, therefore either preventing the body from being infected or decreasing the course of the disease. Tcm and Tem cells are important in effectively controlling infection and avoiding further pathological development [34]. Memory CD8^+^ T cells require Tcm and Tem identification for functional and phenotypic heterogeneity; however, Tem may play more roles in peripheral tissues and has stronger cytotoxic activity than Tcm [35]. Until week 18, the CD4^+^ and CD8^+^ Tcm and Tem cells were still maintained at a high level in the GPS+RBD protein group. These findings suggest that GPS, as an immuno-enhancer, could not only improve the ability of RBD protein to rapidly initiate a cellular immune response but also retain a long-lasting immune memory.

Efficient adjuvants are crucial for improving the immune response of vaccines. Inactivated vaccines and subunit vaccines have weak antigenicity and must be used in conjunction with adjuvants. Although aluminum adjuvants are widely used, they lack the ability to assist antigens in activating cellular immune responses and maintaining long-lasting immune memory [36]. The inactivated COVID-19 vaccines, such as CoronaVac, also have the same problems as mentioned above [37]. However, toll-like receptor (TLR) 7/8 agonist molecule (IMDG) [38] was added to the adjuvant component of Covaxin to enhance the ability of antigen in activating cellular immunity, and the neutralizing antibody levels also rapidly decreased after 6 months of complete immunization [39]. Our research findings indicate that GPS components can assist the SARS-CoV-2 RBD antigen in inducing higher levels of humoral and cellular immune responses and maintaining a more durable immune memory ability.

Many potential adjuvants show promise in vitro but fail to exhibit the same efficacy in vivo. As a traditional Chinese medicine, GPS has many physiological functions, such as enhancing immunity [40]. It has been reported that GPS can inhibit the growth of a transplantable Lewis lung carcinoma (LLC) tumor in C57BL/6 mice, remarkably increasing splenocyte proliferation and the ratio of CD4^+^/CD8^+^ T lymphocytes in peripheral blood in LLC-bearing mice [41]. The clinical research conducted by Cho and his colleagues showed that GPS could enhance immune function, was well tolerated without treatment-related adverse events [42], and could prevent upper respiratory tract infections in clinical trials [43]. Meanwhile, our research also demonstrates that GPS as an adjuvant additive could effectively enhance the humoral and cellular immune response intensity of COVID-19 subunit vaccines. Therefore, it is reasonable to believe that by combining high-throughput sequencing [44] and bioinformatics analysis, researchers will be able to identify the immune enhancement mechanism of GPS and other Chinese herbal extracts more definitely, which could lay the foundation for promoting the application of traditional Chinese medicine adjuvants.

## 5. Conclusions

To summarize, our research shown that GPS could assist SARS-CoV-2 RBD subunit vaccine in establishing stronger systemic and pulmonary mucosal protective immunity and may become a promising vaccine immuno-enhancer. Furthermore, Chinese herbal extracts could be an attractive adjuvant for vaccination, especially TCM polysaccharides.

## Data Availability

The data presented in this study are available upon request from the corresponding author.

## Supplementary Materials

The following supporting information can be downloaded at: https://www.mdpi.com/article/10.3390/vaccines11121833/s1, Figure S1: Preparation of RBD protein and determination of GPS administration; Figure S2: Related to Figure 1, Figure 2 and Figure 4. Induction of Ginseng Polysaccharides on T Cells and Memory B Cells.
